# Comprehensive Secondary Metabolite Profiling and Antioxidant Activity of Aqueous and Ethanol Extracts of *Neolamarckia cadamba* (Roxb.) Bosser Fruits

**DOI:** 10.3390/metabo14090511

**Published:** 2024-09-21

**Authors:** Lin Yang, Liyan Wu, Yongxin Li, Yuhui Yang, Yuting Gu, Jialin Yang, Luzy Zhang, Fanxin Meng

**Affiliations:** 1 School of Pharmacy and Food Science, Zhuhai College of Science and Technology, Zhuhai 519040, Chinawuliyan@zcst.edu.cn (L.W.);; 2 College of Food Sciences, South China Agricultural University, Guangzhou 510642, China; 3 College of Life Science, Jilin University, Changchun 130000, China

**Keywords:** *Neolamarckia cadamba*, *Anthocephalus cadamba*, metabolic profiling, fruit, extraction solvent, bioactivity, LC-MS

## Abstract

Background: *Neolamarckia cadamba* (Rubiaceae) is a well-recognized medicinal plant with recorded therapeutical attributes. However, a thorough assessment of active compounds in its fruits is lacking, limiting their use and valorization in pharmacological industries. Methods: Thus, this study investigated variations in the fruits’ secondary metabolite (SM) profiles, as well as antioxidant activities in aqueous (WA) and ethanol (ET) extracts. Results: Liquid chromatography–electrospray ionization tandem mass spectrometry identified 541 SMs, of which 14 and 1 (di-*O*-glucosylquinic acid) were specifically detected in ET and WA, respectively. Phenolic acids (36.97%), flavonoids (28.10%), terpenoids (12.20%), and alkaloids (9.98%) were the dominant SMs. The SM profiles of the fruits in WA and ET were quite different. We revealed 198 differentially extracted (DE) metabolites between WA and ET, including 62 flavonoids, 57 phenolic acids, 45 terpenoids, 14 alkaloids, etc. Most DE flavones (36 out of 40), terpenoids (45 out of 45), and alkaloids (12 out of 14) had higher content in ET. Catechin and its derivatives, procyanidins, and tannins had higher content in WA. ABTS and DPPH assays showed that the antioxidant activity of ET was significantly higher than that of WA. Conclusions: Our findings will facilitate the efficient extraction and evaluation of specific active compounds in *N. cadamba*.

## 1. Introduction

*N. cadamba,* also referred to as *Anthocephalus cadamba* (Roxb.) Miq., is an economically important evergreen tree member of the Rubiaceae family [1]. It is an underutilized plant native to South and Southeast Asia and was recently introduced in many countries in tropical and subtropical regions [1,2]. Different parts of the plant are widely used to treat numerous ailments, including diabetes, fever, anemia, blood diseases, stomatitis, uterine complaints, leprosy, skin diseases, diverse cancers, eye inflammation, tumors, diarrhea, and infectious diseases [2,3]. Correspondingly, in vitro and in vivo studies demonstrated that the plant possesses diverse therapeutic and biological potentials, including anti-diabetes, antimicrobial, anti-inflammation, wound healing, antioxidation, anthelmintic, anti-cancer, and cardioprotective [2,4,5,6,7,8,9,10,11]. Among the different organs of *N. cadamba*, the fruits have received less attention from researchers, leading to the lack of information on the active compounds in the fruits and their biological properties [2,9,12,13]. Therefore, an effective and comprehensive analysis of bioactive compounds that compose *N. cadamba* fruits will pave the way for mechanistic insights into the therapeutic abilities of the fruits and promote the fruit market and industry.

**Figure 1:**
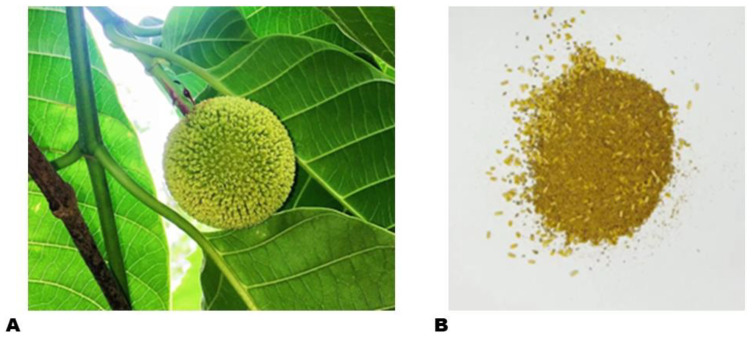
(**A**) A picture of *Neolamarckia cadamba* fruit. (**B**) Fine powder of the fruits, which were dried and pulverized.

*N. cadamba* flowering and fruiting times vary depending on local climatic and soil conditions, and the fruits are procurable across the year in one region or others of the world [13]. The fruits occur in small and pulpy capsules that are crowded together into a fleshy yellow-orange ball containing multiple seed coats (Figure 1) [13]. Previous phytochemical investigations showed that *N. cadamba* fruits contain a diversity of compounds, including flavonoids, tannins, terpenoids, alkaloids, and phenolic acids [7,12,14,15,16]. However, a thorough and extensive examination of the secondary metabolite (SM) profile of the fruits is lacking, which limits knowledge of key and important bioactive compounds. Plant SMs, including alkaloids, flavones, anthocyanins, terpenoids, flavonols, proanthocyanins, phenolic acids, tannins, etc., are vital phytomedicines, with preventive and curative effects against human chronic and lifestyle diseases [17,18,19,20,21,22,23]. With regard to wide distribution and importance in traditional medicine, a comprehensive characterization of the *N. cadamba* fruit SM profile will enhance the consumption and quality values of the fruits and trigger their valorization in the food pharmacy and diverse industries.

Metabolomics analytical technologies are essential components of modern systems biotechnology [24]. They are used to assess and monitor the nutritional quality of vegetables, crops, and fruits [24,25,26]. The application of metabolomics in fruit trees has recently expanded, and many mechanisms, such as fruit quality formation and variation, biotic/abiotic stress responses of fruit plants, metabolic modulation of fruit traits, etc., are explored [24]. Four different approaches, including targeted metabolomics, pseudo-targeted metabolomics, untargeted metabolomics, and widely targeted metabolomics, and various analytical methods, such as liquid chromatography (LC), nuclear magnetic resonance (NMR), mass spectrometry (MS), and gas chromatography (GC), are used in metabolomics [24,27,28,29]. Among the different metabolomics approaches, widely targeted metabolite profiling is used to explore the global metabolome of plant-derived products, resulting in an accurate qualitative and quantitative identification of a wide range of metabolites of interest and a thorough understanding of metabolic diversity and pathways in plants [30,31,32,33]. A metabolomics experiment routinely comprises four steps: (i) samples’ collection, (ii) extraction, (iii) analysis of the extract, and (iv) data reduction and statistical analyses. Generally, extraction is the most critical step, and it has considerable effects on the interpretation of results [34]. Solely a repeatable extraction method with good efficiency may generate reliable data since the metabolites to be identified and chemically characterized are the ones that solubilize in the solvent [34,35]. The solvent dependency of the sample composition has a profound impact on the analysis of the sample by either chromatography-based or gravimetric methods in accuracy, precision, and mass balance [36]. Accordingly, it is of great research interest to evaluate the metabolite profiles of different extracts of *N. cadamba* fruits.

The primary aim of this present study was to comprehensively analyze the SM profiles of aqueous (WA) and ethanol (ET) extracts of *N. cadamba* fruits and explore the impacts of the extraction processes on the antioxidant capacities of the fruits. Although some previous studies used methanol as the extraction solvent, we did not include it due to its high toxicity and the health risks associated with its use [37]. We hypothesized that the two extraction solvents (ET and WA) might be effective for some categories of SMs, respectively. Ultra-performance liquid chromatography–mass spectroscopy-based broadly targeted metabolomics was applied, and differentially extracted metabolites were revealed. Our findings will promote research on active compounds in *N. cadamba* fruits. 

## 2. Materials and Methods

### 2.1. Plant Material

The fruits were harvested in December 2020 at the Planting Base located in the Daoxiang Garden of South China Agricultural University. They were then placed in a cool and ventilated place and later dried in an oven at 50 °C (Figure 1). Next, the fruits were crushed and sieved with a 40 mesh. The powder was sealed and stored at room temperature for a period of less than two weeks. All the experiments were repeated three times.

### 2.2. Aqueous and Ethanolic Extractions

The aqueous extract was obtained by the water-boiling extraction method. Mixed fruit powder and distilled water (ratio of 1:25) were evenly stirred and left to stand for 30 min [38]. Then, the mixture was boiled for 30 min [38]. After centrifugation (5000× *g* for 20 min), the residue was re-extracted, and the two collected supernatants were pooled together for the next step. For the ethanol extraction, fruit powder and 70% ethanol in a 1:25 ratio were mixed and placed in a shaker (200 r/min) at 37 °C for 72 h [38]. Then, the samples were centrifugated, and the supernatants were collected. Both the aqueous and ethanolic extracts were subjected to rotary evaporation, and the solutions were concentrated to 1/5 of the original volume. Finally, a vacuum freeze-drying machine was used to transform the concentrated extracts into dry powders that were stored at 4 °C.

### 2.3. Metabolite Extraction and UPLC-MS/MS Analysis

An amount of 100 mg of the extracts was dissolved in 1.2 mL of 70% methanol. Subsequently, the extracts were filtrated (0.22 μm micropore membrane SCAA-104, ANPEL, Shanghai, China) and stored at −20 °C. The UPLC-ESI-QqQLIT-MS/MS analysis was performed at Metware Biotechnology Co., Ltd., Wuhan, China [32,33,39,40]. Equal amounts of aqueous and ethanolic extracts were mixed to generate QC (mix, quality control). The metabolomics conditions were as described previously [33,39,40]. The specific LC and MS conditions are described in Table S1.

The mass spectra information (Q1 and Q3 values, collision energy, fragmentation patterns, and de-clustering potential) and retention times were compared to the standards for qualitative metabolite identification. In addition, the metabolites were structurally confirmed based on the in-house database (Metware Biotechnology Co., Ltd.) and search in open databases (MassBank, MoTo DB, HMDB, KNApSAcK, and METLIN) [32,39]. The relative contents of metabolites were calculated via the MRM mode with SCIEX-OS software (version 1.4). 

### 2.4. Metabolomics Data Analysis

The multivariate analyses were carried out in R (version 4.1.0). The R packages pheatmap, cor, MetaboAnalystR, and prcomp were, respectively, utilized for hierarchical clustering analysis, correlation analysis, orthogonal partial least squares discriminant analysis, and principal component analysis. Differentially extracted metabolites (DEMs) were detected using ggplot2 program in R. The applied thresholds were Log2FC ˃ 1, *p*-value < 0.05, and VIP ≥ 1. The variable important in the projection (VIP) values were filtered out from the OPLS-DA results. Excel 2016 and GraphPad Prism (v8.0.01, La Jolla, CA, USA) were used for data processing and graph construction. TBtools was utilized to construct Venn diagrams and heatmaps [41].

### 2.5. Antioxidant Activity Tests

For the antioxidant and antibacterial tests, the fruits’ aqueous and ethanol extracts were dissolved, respectively, in distilled water to obtain test solutions of 100 mg/mL. The antioxidant activities were evaluated through the DPPH (2,2-diphenyl-1-picrylhydrazyl) and ABTS (2,2′-azino-bis (3-ethylbenzothiazoline-6-sulfonic acid) assays following the methods described by Dossou et al. [33].

## 3. Results

### 3.1. Secondary Metabolite Profiles of N. Cadamba Fruits in Aqueous and Ethanolic Extracts

**Figure 2:**
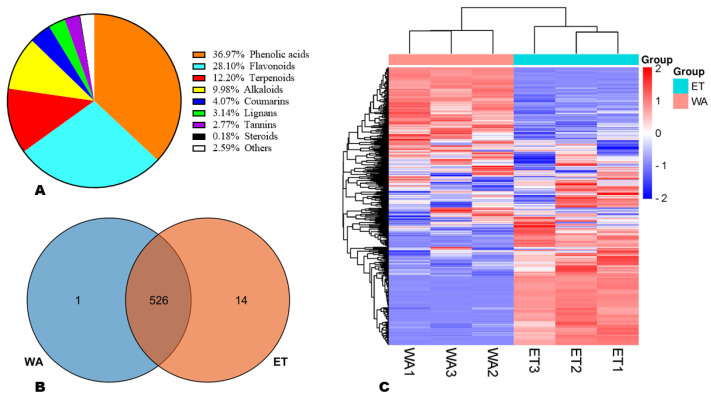
Diversity and variation in secondary metabolite profiles in aqueous (WA) and 70% ethanol (ET) extracts of *N. cadamba* fruits. (**A**) Classification of the 541 identified secondary metabolites; (**B**) Venn diagram showing the common number of metabolites identified in WA and ET; (**C**) hierarchical clustering analysis (HCA).

To identify an appropriate solvent for the efficient extraction of different categories of secondary metabolites (SM) in *N. cadamba* fruits (Figure 1), we carried out a comparative widely targeted SM profiling of the aqueous (WA) and 70% ethanol (ET) extracts of the fruits. The detection of metabolites in the extracts operated at both the positive and negative electrospray ionization and the ion chromatograms of some identified compounds in the quality control (QC) samples are shown in Figure S1. The QC samples showed very high correlations (r ≥ 0.99), confirming the repeatability of the experiment (Figure S2A). Totally, we structurally identified 541 SMs, including 36.97% phenolic acids, 28.10% flavonoids, 12.20% terpenoids, 9.98% alkaloids, 4.07% coumarins, 3.14% lignans, 2.77% tannins, 1 steroid, and others (Figure 2A, Table S2). One phenolic acid (di-*O*-glucosylquinic acid) was specifically detected in WA (Figure 2B, Table S2). Meanwhile, fourteen metabolites, including six phenolic acids (5-*O*-*p*-coumaroylquinic acid, idescarpin, 1-caffeoylquinic acid, 2-*O*-caffeoylmalic acid, chlorogenic acid methyl ester, and 2′,6′-dihydroxyacetophenone), two flavonoids (puerarin-4′-*O*-glucoside and quercetin-3-*O*-(2″-*O*-galactosyl)glucoside), two terpenoids (deacetylasperulosidic acid and ursonic acid), one alkaloid (4-coumaroylcholine), one coumarin (Fraxetin), and two unclassified metabolites (3,5,7,4′-tetrahydroxy-coumaronochromone and senkyunolide A) were specifically identified in ET (Figure 2B, Table S2).

In order to explore the variability in the fruit’s SM profiles in WA and ET, we conducted HCA (hierarchical clustering analysis) and correlation analyses (Figure 2C and Figure S2B). As shown in Figure 2C, the HCA plot showed that the fruit’s SM profiles in WA and ET were very different. Some metabolites exhibited higher relative contents in WA, while others showed a higher relative content in ET (Figure 2C). Interestingly, the correlation analysis result was supportive of the HCA (Figure S2B). 

### 3.2. Differential Accumulation of Metabolites in Aqueous and Ethanolic Extracts

**Figure 3:**
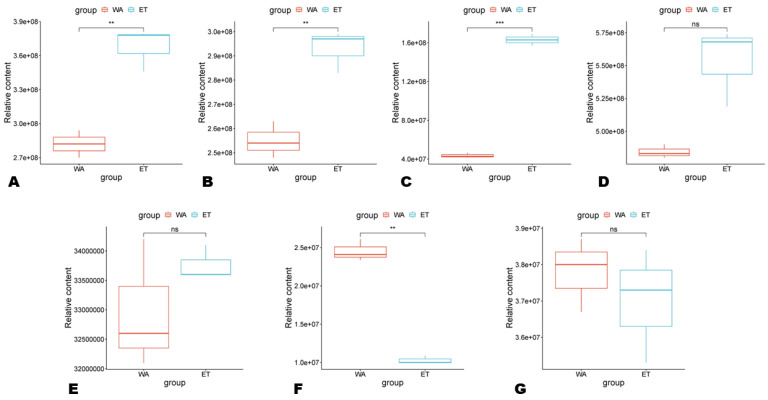
Accumulation patterns of secondary metabolites in aqueous (WA) and ethanolic extracts. (**A**) Alkaloids; (**B**) flavonoids; (**C**) terpenoids; (**D**) phenolic acids; (**E**) lignans; (**F**) tannins; (**G**) coumarins. ns, non-significant. **, and *** indicate statistically different at *p* ˂ 0.01, and 0.001, respectively.

To investigate the variation in the extraction of SMs in WA and ET, we calculated and compared the sum of the relative levels of all metabolites within each class (Figure 3). The results showed that ET yielded significantly higher alkaloids, flavonoids, and terpenoids than WA (Figure 3A–C). In contrast, WA yielded significantly higher tannins than ET (Figure 3F). Although the relative contents of phenolic acids and lignans in ET were higher than in WA, the differences were insignificant (Figure 3D,E). Meanwhile, there was no significant difference in the relative content of coumarins between WA and ET (Figure 3G).

### 3.3. Differentially Extracted Metabolites (DEMs) between Aqueous and Ethanolic Extracts 

**Figure 4:**
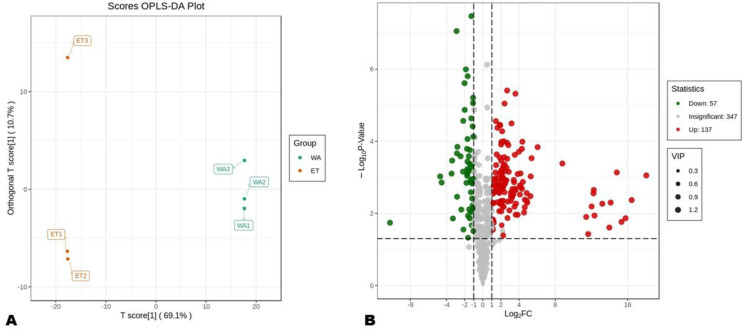
Differentially extracted metabolites (DEMs) between aqueous and ethanolic extracts. (**A**) OPLS-DA score plot of pairwise comparisons between WA and ET; (**B**) volcano plot of DEMs in pairwise comparisons between WA and ET.

To reveal the metabolites that were differentially extracted in WA and ET, we conducted an OPLS-DA analysis. The OPLS-DA score plot confirmed that the SM profiles of the fruits in WA and ET were very different (Figure 4A). The permutation plot shows that the R^2^Y of the pairwise comparison between WA and ET was equal to 1, and the Q^2^ was equal to 0.991, confirming the model was reliable (Figure S3). By applying the thresholds of VIP ≥ 1, Log_2_FC > 1, and *p*-value < 0.05, we revealed a total of 198 DEMs between WA and ET (Figure 4B). Of those, 137 were highly extracted in ET DAMs (Figure 4B). 

#### 3.3.1. Differentially Extracted Flavonoids

**Figure 5:**
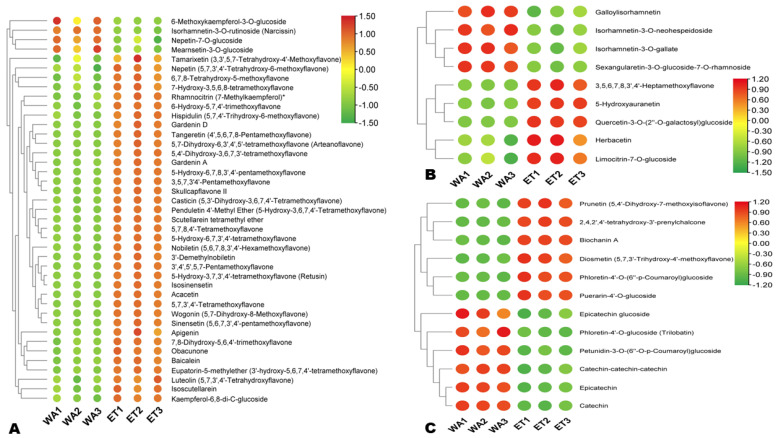
Differentially extracted flavonoids between aqueous (WA) and ethanolic extracts (ET). (**A**) Flavones; (**B**) flavonols; (**C**) other flavonoids.

In total, 62 flavonoids, including 41 flavones, 9 flavonols, 3 isoflavones, 1 anthocyanin, 3 chalcones, 4 flavanols, and 1 flavanone were differentially extracted between WA and ET (Figure 5A–C). Of those, 14 and 48 were highly extracted in WA and ET, respectively (Figure 5A–C). Quercetin-3-*O*-(2″-*O*-galactosyl)glucoside, puerarin-4′-*O*-glucoside, kaempferol-6,8-di-C-glucoside, obacunone, 2,4,2′,4′-tetrahydroxy-3′-prenylchalcone, 6-hydroxy-5,7,4′-trimethoxyflavone, 5-hydroxyauranetin, 5-hydroxy-6,7,8,3′,4′-pentamethoxyflavone, and 7,8-dihydroxy-5,6,4′-trimethoxyflavone were the most highly extracted flavonoids in ET with a Log2FC of 15.32, 11.64, 3.30, 2.94, 2.86, 2.68, 2.67, 2.62, and 2.47, respectively (Figure 5A–C). Catechin-catechin-catechin (|Log2FC| = 2), epicatechin (|Log2FC| = 2.81), and catechin (|Log2FC| = 2.85) were the most highly extracted flavonoids in WA (Figure 5C).

#### 3.3.2. Differentially Extracted Phenolic Acids and Terpenoids

**Figure 6:**
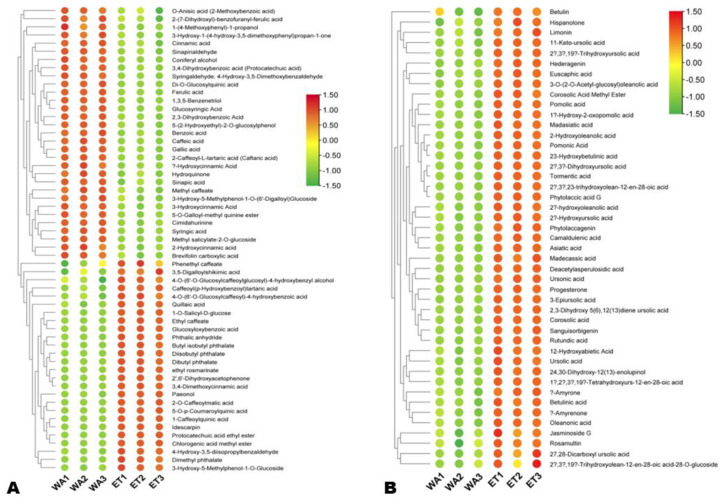
Differentially extracted phenolic acids (**A**) and terpenoids (**B**) between aqueous (WA) and ethanolic extracts (ET).

In total, 57 phenolic acids (including 26 highly extracted in ET) and 45 terpenoids (all highly extracted in ET) were identified (Figure 6A,B). Of the phenolic acids, 1-caffeoylquinic acid, idescarpin, 5-*O*-*p*-coumaroylquinic acid, chlorogenic acid methyl ester, 2-*O*-caffeoylmalic acid, 2′,6′-dihydroxyacetophenone, paeonol, protocatechuic acid ethyl ester, caffeoyl(p-hydroxybenzoyl)tartaric acid, 1-*O*-salicyl-D-glucose, and glucosyloxybenzoic acid were the most highly extracted in ET, with a Log2FC of 18.05, 16.44, 15.76, 14.79, 14.12, 12.33, 8.78, 6.05, 5.38, 5.29, and 5.26, respectively (Figure 6A). In WA, 2-caffeoyl-L-tartaric acid (caftaric acid, |Log2FC| = 3.38), glucosyringic acid (|Log2FC| = 3.45), cimidahurinine (|Log2FC| = 4.55), 5-(2-hydroxyethyl)-2-*O*-glucosylphenol (|Log2FC| = 4.71), and di-*O*-glucosylquinic acid (|Log2FC| = 10.25) were the most highly extracted phenolic acids (Figure 6A). The top differentially extracted terpenoids in ET included ursonic acid (|Log2FC| = 13.97), deacetylasperulosidic acid (|Log2FC| = 12.25), 2-hydroxyoleanolic acid (|Log2FC| = 4.91), 3-epiursolic acid (|Log2FC| = 4.85), 2α-hydroxyursolic acid (|Log2FC| = 4.84), pomonic Acid (|Log2FC| = 4.64), oleanonic acid (|Log2FC| = 4.63), and 24,30-dihydroxy-12(13)-enolupinol (|Log2FC| = 4.55) (Figure 6B).

#### 3.3.3. Differentially Extracted Alkaloids, Proanthocyanins, Tannins, Lignans, and Coumarins

**Figure 7:**
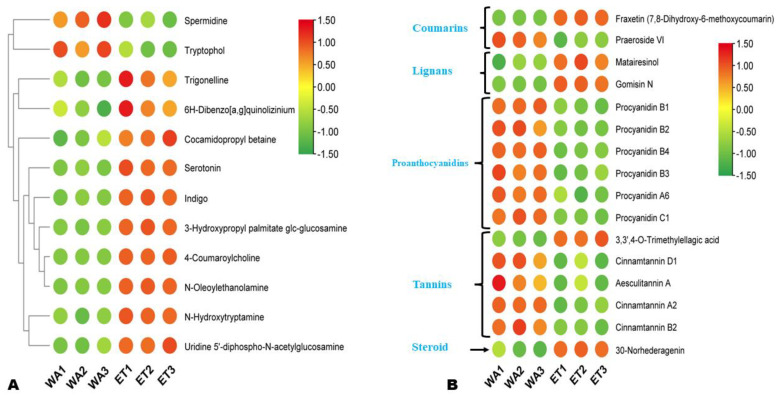
Differentially extracted alkaloids (**A**) and other secondary metabolites (**B**) between aqueous (WA) and ethanolic extracts (ET).

Twelve differentially extracted alkaloids were identified, including ten highly extracted in ET (Figure 7A). Of those, 4-coumaroylcholine, N-oleoylethanolamine, 3-hydroxypropyl palmitate glc-glucosamine, and indigo were the top differentially extracted in ET, with a Log2FC of 12.22, 4.17, 3.62, and 3.61, respectively. Regarding the other classes of DEMs, fraxetin (coumarin), gomisin N (lignan), and 30-norhederagenin (steroid) were the most highly extracted in ET, with a Log2FC of 12.01, 3.37, and 3.17, respectively (Figure 7B). Meanwhile, praeroside VI (|Log2FC| = 3.3), procyanidin B1 (|Log2FC| = 2.45), and procyanidin B4 (|Log2FC| = 2.02) were the top highly extracted in WA (Figure 7B).

### 3.4. Variation in Antioxidant Activities of Aqueous and Ethanolic Extracts of N. cadamba Fruits

**Figure 8:**
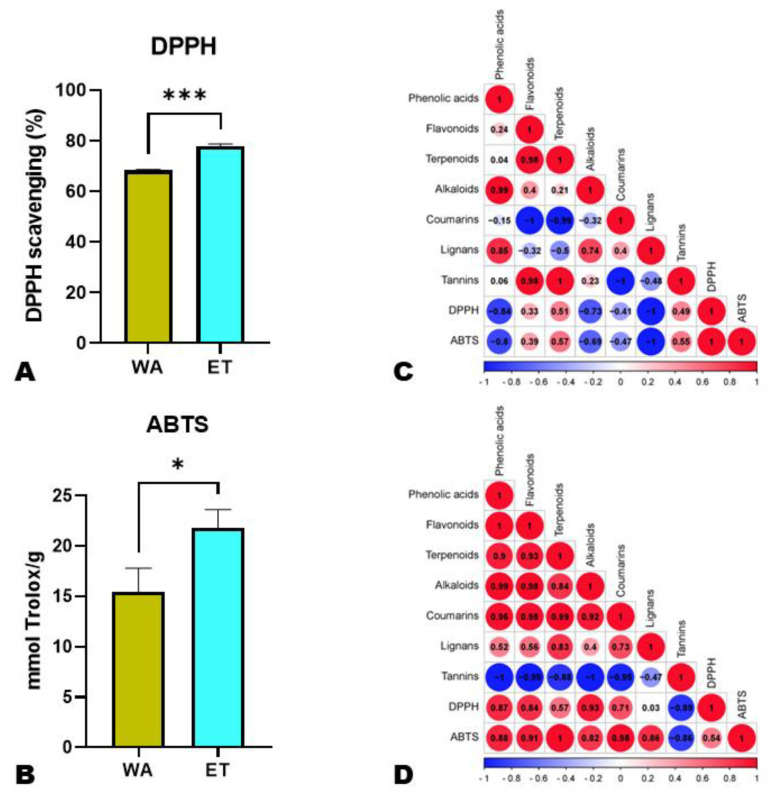
Antioxidant activities of aqueous (WA) and ethanolic (ET) extracts of *N. cadamba* fruits. (**A**) DPPH essay; ABTS (**B**) essay; (**C**) correlations between antioxidant activity and SM classes in WA; (**D**) correlations between antioxidant activity and SM classes in ET. * and *** indicate statistically different at *p* ˂ 0.05 and 0.001, respectively.

To verify whether the extraction solvent would affect the biological activities of *N. cadamba* fruits, we performed antioxidant activity tests. As shown in Figure 8A,B, the antioxidant activities of ET were significantly higher than those of WA. All SMs showed significant positive correlations with the antioxidant activity (DPPH and ABTS) in ET, except for tannins (Figure 8D). In contrast, in WA, only tannins, flavonoids, and terpenoids had positive correlations with the antioxidant activities. 

## 4. Discussion

*N. cadamba* fruits are promising materials for high pharmacological applications and economic values. However, the fruit has received less research attention compared to leaves, roots, and bark, and a deep understanding of its quality values is lacking [2,13]. Hence, a global analysis and detailed information on the SMs that compose the fruit are of general interest. The present study comprehensively revealed the SM profile of *N. cadamba* fruits, providing an overview of the diversity of bioactive phytochemicals. We confidentially identified 541 SMs, of which phenolic acids, flavonoids, terpenoids, and alkaloids accounted for the large part. These results indicate that they are the major bioactive compounds in *N. cadamba* fruits and may represent critical characteristics in evaluating the fruits’ quality. Moreover, they suggest that *N. cadamba* fruits may own various biological and therapeutic attributes as per the other organs, including leaves, bark, and roots. Flavonoids, terpenoids, and alkaloids possess remarkable health-promoting abilities, including anti-cancer, antioxidation, anti-diabetes, anti-inflammatory, anti-angiogenic, anti-neuroinflammation, anti-insomnia, anti-influenza, anti-amnesia, and anti-osteoclastogenesis [21,22,42,43,44,45,46,47,48,49,50]. For instance, compounds such as diosmetin, apigenin, luteolin, hispidulin, nepetin, kaempferol, and various catechins have been proven to relieve lifestyle diseases, including cancer, inflammation, oxidation diseases, neuropathologies, diabetes, infections, osteoclastogenesis, etc. [43,46,51,52,53,54,55,56,57]. Consistent with this, some previous pharmacological investigations on the fruits showed that they possess antioxidant, anthelmintic, anti-diabetic, and membrane-stabilizing properties [2,7,12,14,15]. With this metabolic exposure, further pharmacological studies are needed to ascertain the therapeutical potentials of the fruits and identify the key active compounds for quality improvement and industrial purposes. In addition, integrated omics tools should be applied to decipher the molecular regulation of flavonoid, alkaloid, phenylpropanoid, and terpenoid biosynthesis in developing *N. cadamba* fruits. With the availability of *N. cadamba* genome data [1], this exposure to the fruit’s SM profile may trigger quality-related gene mining and the dissection of gene–metabolite interactions. 

The extraction step is the most critical during metabolomics experiments [34]. Any defects in the extraction process often result in inaccurate data interpretation and conclusions [34]. Ideally, the extracts to be analyzed should contain all the intact metabolites at a detectable concentration [34]. Usually, different solvents give rise to different metabolome profiles of the same sample [34,35]. Concordantly, we found that the SM profiles of *N. cadamba* fruits in WA and ET differed significantly. One phenolic acid (di-*O*-glucosylquinic acid) was specifically identified in WA, as well as fourteen other metabolites in ET. The relative contents of flavonoids, terpenoids, and alkaloids were significantly higher than in WA. Meanwhile, the relative content of tannin was significantly higher in WA than in ET. Supportively, most differentially extracted flavones, alkaloids, and terpenoids (all) had higher contents in ET, while all differentially extracted catechin and its derivative, procyanidins, and tannins exhibited the highest contents in WA. The high solubility of flavonoids, alkaloids, and terpenoids in ET shows that these classes of metabolites in *N. cadamba* fruit are less soluble in aqueous solutions. It has been demonstrated that most polyphenol compounds have a higher solubility index in organic solvents than in aqueous solvents [58,59]. Similarly, Cuevas-Valenzuela et al. have shown that catechin and its derivatives are more soluble in water than in organic solvents [60]. These results show that ethanol might be efficient for analyzing flavonoid, alkaloid, and terpenoid compounds in *N. cadamba* fruits. Meanwhile, distilled water might be the most suitable for analyzing catechin and its derivatives, procyanidins, and tannins. Although some previous studies used methanol as the extraction solvent, we did not include it due to its high toxicity and the health risks associated with its use [37]. The choice of solvents for extraction is a marriage between their bulk physicochemical properties and their capability to dissolve compounds because they possess complementary intermolecular interactions with the target compounds [61]. Further considerations include purity, mutual solubility, availability, health and safety issues, and waste management [61]. 

The boiling step during the aqueous extraction could have also affected the SM profile of the fruits, as some substances may have degraded or converted into other compounds at 100 °C. The migration or release of extractables from a material sample into a solvent is both a thermodynamically (solubility) and kinetically (diffusion) driven process [36]. It depends on extraction-related factors (solvent, temperature, contact time, etc.) and material-related factors (amount of available migratable compounds in a material, the free volume of the material, etc.) [62]. The observed great SM profile variation in the fruits in ET and WA may depend upon the diffusion coefficient and partition coefficient attributed to the above factors, including the solvent (nature, polarity, solubility, etc.), extraction process, and sample–solvent intermolecular interactions [36,61]. Future studies need to investigate other conventional and modern extraction methods [63].

Plant SMs play essential roles in delaying or inhibiting lipid peroxidation by functioning as scavengers that preclude the expansion of oxidative chains [64]. Accordingly, the consumption of fruit or fruit products rich in SMs helps prevent many long-lasting diseases, such as obesity, stroke, cancers, diabetes, hypertension, etc. [64]. The significantly higher flavonoid, alkaloid, and terpenoid contents of ET show that the ethanolic extracts of *N. cadamba* fruits might possess a higher antioxidant capacity than WA. Consistently, the ABTS and DPPH assays revealed significantly higher antioxidant activities of ET over WA. In addition, all the identified SMs except for tannins exhibited significant positive correlations with the antioxidant activity in ET. The high antioxidant capacity of phenolic compounds has been proven [65]. These results denote that ethanol might be the appropriate solvent for the effective extraction of antioxidation-related compounds from *N. cadamba* fruits for pharmacological applications. These active substances can be efficiently extracted and subjected to bioavailability, toxicity, in vivo health effects, and mechanisms investigations. Antioxidant assessments in vitro and in vivo are primarily used to determine the antioxidant capacity in diverse biological and food samples [66]. Unfortunately, there are non-comparable results and untrustworthy information due to the lack of method and interpretation standardization [66]. Therefore, these in vitro antioxidant results could inform the high antioxidant capacity of *N. cadamba* fruits. However, the DPPH assay does not evaluate the characteristics of antioxidants and does not necessarily show the capacity to suppress oxidation; it is recommended that these extracts be further assessed for their effect on the levels of plasma lipid peroxidation in vitro and biomarkers of oxidative stress in vivo [67].

## 5. Conclusions

This study revealed the differences in secondary metabolite profiles, as well as the antioxidant and antibacterial activities of *N. cadamba* fruits in aqueous and ethanol extracts. One phenolic acid (di-*O*-glucosylquinic acid) was detected specifically in WA and fourteen metabolites (5-*O*-*p*-coumaroylquinic acid, idescarpin, 1-caffeoylquinic acid, 2-*O*-caffeoylmalic acid, chlorogenic acid methyl ester, 2′,6′-dihydroxyacetophenone, puerarin-4′-*O*-glucoside, quercetin-3-*O*-(2″-*O*-galactosyl)glucoside, deacetylasperulosidic acid, ursonic acid, 4-coumaroylcholine, fraxetin, 3,5,7,4′-tetrahydroxy-coumaronochromone, and senkyunolide A) were specifically detected in ET. We uncovered 198 DEMs and exposed their extraction patterns in WA and ET. Flavonoids, alkaloids, and terpenoids had significantly higher contents in ET than in WA. Accordingly, ethanol is recommended for the efficient extraction of flavones, alkaloids, and terpenoids from *N. cadamba* fruits. Similarly, distilled water should be used to analyze catechin and its derivatives, procyanidins, and tannins. Compared to WA, ET showed a significantly higher antioxidant capacity, indicating that ethanol is effective for extracting antioxidant-related compounds from *N. cadamba* fruits. Our results may facilitate the efficient extraction and biological assessment of active compounds in *N. cadamba*. Notably, our findings represent guidelines for the targeted analysis of specific bioactive compounds in *N. cadamba* fruits.

## Data Availability

The data supporting the reported results are included in this manuscript and its Supplementary Files. The datasets analyzed or generated will be made available on request.

## Supplementary Materials

The following supporting information can be downloaded at: https://www.mdpi.com/article/10.3390/metabo14090511/s1, Figure S1: multiple reaction monitoring (MRM) graphs of QC samples showing total ion current (TIC) of some identified metabolites; Figure S2: correlation analysis plot of samples; Figure S3: permutation plot of pairwise OPLS-DA analysis between WA and ET; Table S1: liquid chromatography (A) and mass spectrometry (B) conditions; Table S2: list of the 541 identified secondary metabolites and their relative contents.
