# Supplementary material for: Comprehensive Secondary Metabolite Profiling and Antioxidant Activity of Aqueous and Ethanol Extracts of Neolamarckia cadamba (Roxb.) Bosser Fruits
Source: Metabolites. 2024 Sep 21;14(9):511. doi: 10.3390/metabo14090511 (PMC11434403; doi:10.3390/metabo14090511)
Supplement: Supplementary file 1 [file metabolites-14-00511-s001.zip › Supplementary Figures.pptx]

## Slide 1
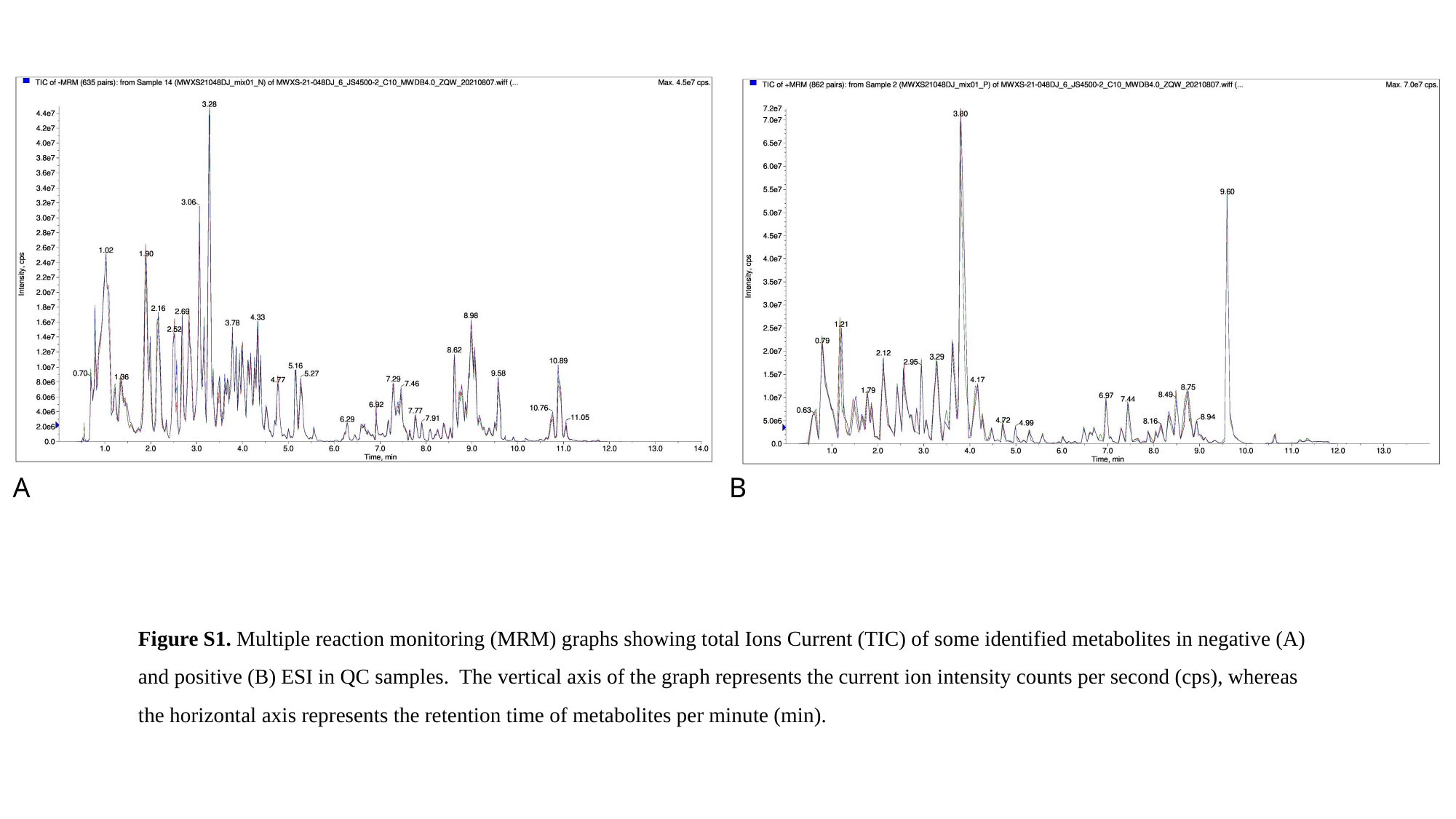

A
B
Figure S1. Multiple reaction monitoring (MRM) graphs showing total Ions Current (TIC) of some identified metabolites in negative (A) and positive (B) ESI in QC samples. The vertical axis of the graph represents the current ion intensity counts per second (cps), whereas the horizontal axis represents the retention time of metabolites per minute (min).

## Slide 2
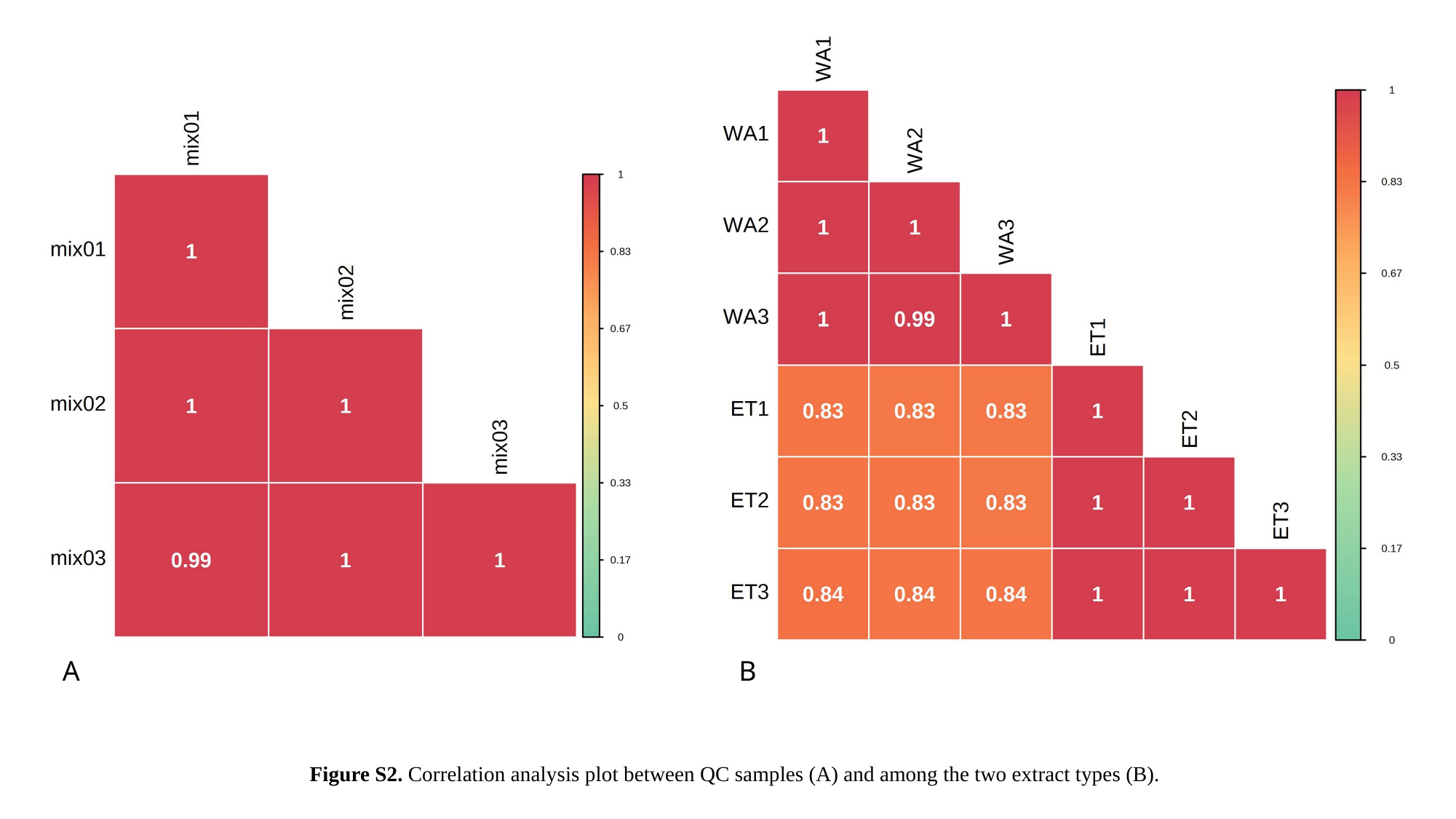

A
B
Figure S2. Correlation analysis plot between QC samples (A) and among the two extract types (B).

## Slide 3
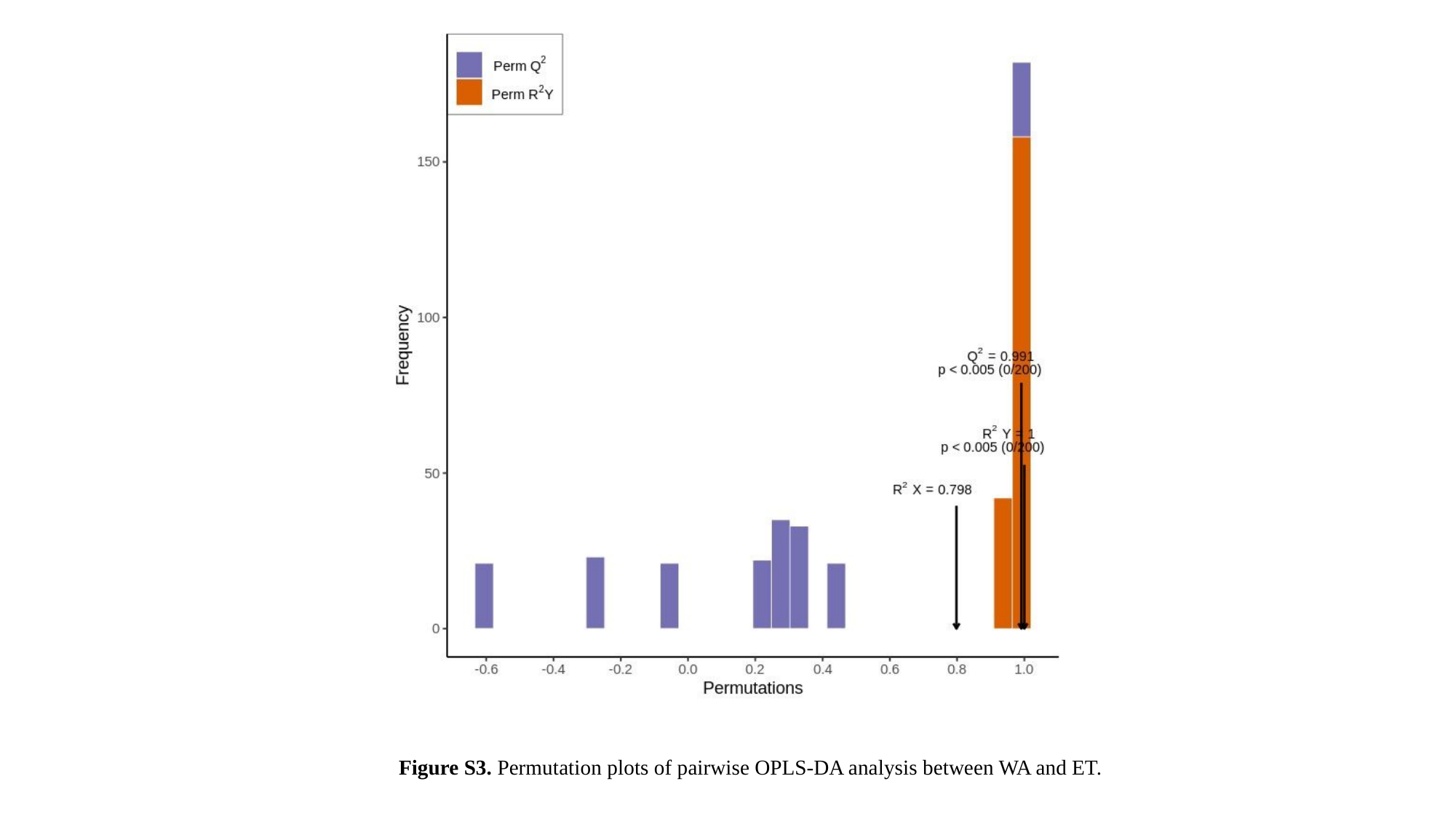

Figure S3. Permutation plots of pairwise OPLS-DA analysis between WA and ET.
